# Modulation of blood pressure regulatory genes in the *Agtrap‐Plod1* locus associated with a deletion in *Clcn6*

**DOI:** 10.14814/phy2.15417

**Published:** 2022-08-04

**Authors:** Christine A. Klemens, Lashodya V. Dissanayake, Vladislav Levchenko, Adrian Zietara, Oleg Palygin, Alexander Staruschenko

**Affiliations:** ^1^ Department of Molecular Pharmacology and Physiology University of South Florida Tampa Florida USA; ^2^ Hypertension and Kidney Research Center University of South Florida Tampa Florida USA; ^3^ Department of Physiology Medical College of Wisconsin Milwaukee Wisconsin USA; ^4^ Department of Medicine Medical University of South Carolina Charleston South Carolina USA; ^5^ James A. Haley Veterans' Hospital Tampa Florida USA

**Keywords:** ANP, CLCN6, MTHFR, NPPA, NPPB, Stroke

## Abstract

The AGTRAP‐PLOD1 locus is a conserved gene cluster containing several blood pressure regulatory genes, including *CLCN6*, *MTHFR*, *NPPA*, and *NPPB*. Previous work revealed that knockout of *Clcn6* on the Dahl Salt‐Sensitive (SS) rat background (SS‐*Clcn6*) resulted in lower diastolic blood pressure compared to SS‐WT rats. Additionally, a recent study found sickle cell anemia patients with mutations in *CLCN6* had improved survival and reduced stroke risk. We investigated whether loss of *Clcn6* would delay the mortality of Dahl SS rats on an 8% NaCl (HS) diet. No significant difference in survival was found. The ability of *Clcn6* to affect mRNA expression of nearby *Mthfr, Nppa*, and *Nppb* genes was also tested. On normal salt (0.4% NaCl, NS) diets, renal *Mthfr* mRNA and protein expression were significantly increased in the SS‐*Clcn6* rats. MTHFR reduces homocysteine to methionine, but no differences in circulating homocysteine levels were detected. *Nppa* mRNA levels in cardiac tissue from SS‐*Clcn6* rat in both normotensive and hypertensive conditions were significantly reduced compared to SS‐WT. *Nppb* mRNA expression in SS‐*Clcn6* rats on a NS diet was also substantially decreased. Heightened *Mthfr* expression would be predicted to be protective; however, diminished *Nppa* and *Nppb* expression could be deleterious and by preventing or blunting vasodilation, natriuresis, and diuresis that ought to normally occur to offset blood pressure increases. The conserved nature of this genetic locus in humans and rats suggests more studies are warranted to understand how mutations in and around these genes may be influencing the expression of their neighbors.

## Graphical abstract

In this study, we tested whether loss of *Clcn6* would be protective against high salt‐induced mortality. While we observed no significant differences in longevity or neuronal cell death between the SS‐*Clcn6* knock‐out and SS‐WT groups, we found that a mutation in *Clcn6* could be associated with changes to mRNA expression of nearby *Mthfr*, *Nppa*, and *Nppb* genes, thereby complicating the genetic phenotype of our knockout animal.

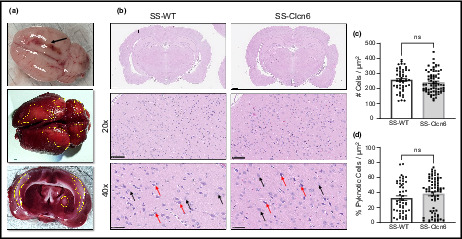

## 1 INTRODUCTION

Increased stroke risk resulting from high blood pressure (BP) is well established, as hypertension results in cerebral artery hypertrophy and remodeling, reductions in endothelial‐dependent relaxation, and impaired autoregulation, among other pathological changes. Genetic variants, such as those in *CLCN6* that reduce BP may also be expected to lower the risk of stroke within this population (Flister et al., 2013; Pereira et al., 2015; Tomaszewski et al., 2010; Zhang et al., 2015). A recent paper found that loss of function mutations in *CLCN6* were associated with a “long survivor” group of Cameroonian sickle cell anemia patients (Wonkam et al., 2020). The median survival of this patient cohort was 44 years old, compared to the “overt stroke” cohort, which had a median age of 20.5 years (Wonkam et al., 2020). *CLCN6* encodes the voltage‐sensitive chloride channel, ClC‐6 (Brandt & Jentsch, 1995). ClC‐6 is an intracellular protein with both channel and transporter characteristics and is expressed in a broad range of tissues, including brain, vascular smooth muscle, and kidney (Brandt & Jentsch, 1995; Buyse et al., 1998; Ignoul et al., 2007; Klemens et al., 2021; Lamb et al., 1999; Neagoe et al., 2010). Previous work from our group demonstrated that a functional knockout of *Clcn6* on a Dahl Salt‐Sensitive (SS) rat background had significantly reduced diastolic blood pressure in normotensive and hypertensive conditions and altered vascular smooth muscle cell function (Klemens et al., 2021). Additionally, the AGTRAP‐PLOD1 locus gene cluster where *CLCN6* is localized has been associated with stroke along with other BP regulatory genes such as *MTHFR, NPPA*, and *NPPB*, (Del Greco et al., 2011; Flister et al., 2013; Pereira et al., 2015; Yang et al., 2014).

Of particular interest is *MTHFR*, which encodes methylenetetrahydrofolate reductase (MTHFR), an enzyme in homocysteine metabolism linked to the folate cycle. Mutations in *MTHFR* have been linked to increased cardiovascular and stroke risk and circulating homocysteine levels (Kumar et al., 2015; Moll & Varga, 2015; Song et al., 2016). Homocysteine is an amino acid derivative of methionine that is either excreted by the kidneys or recycled for remethylation to methionine or transsulfuration to cystathionine. The genetic mutations disrupting this system are associated with blood coagulation and vascular disease (Froese et al., 2016; Selhub, 1999). *MTHFR* and *CLCN6* are located in a head‐to‐head orientation with translational start codons only about 3 kb apart (Gaughan et al., 2000). Furthermore, this region is in linkage disequilibrium and contains several SNPs in haplotype blocks that have been associated with BP control. This region includes a SNP that falls in a transcriptionally active region of both genes and is predicted to alter *MTHFR* and *CLCN6* expression (Del Greco et al., 2011; Flister et al., 2013; Tomaszewski et al., 2010). The inter‐relatedness *MTHFR* and *CLCN6* expression and function, however, has not been investigated.

In addition to *MTHFR*, *NPPA* and *NPPB*, which encode the atrial natriuretic peptide (ANP) and natriuretic peptide B (BNP), respectively, are also located in this complex regulatory genetic landscape. Elevated ANP, BNP, and the N‐terminal portion of its prohormone (NT‐proBNP), are prognostic indicators of ventricular dysfunction and heart failure. The polymorphisms in and around these genes are also associated with altered ANP, BNP, and NT‐proBNP levels (Maalouf & Bailey, 2016; Pereira et al., 2015; Song et al., 2015; Xhaard et al., 2022). Release of ANP and BNP from the heart under high blood volume and ventricular pressure acts as a brake for the Renin‐Angiotensin‐Aldosterone‐System (RAAS) by initiating vasodilation and increasing Na^+^ and water excretion in the kidney. It has also been previously demonstrated that genetic delivery of ANP can reduce stroke mortality in Dahl SS rats (Lin et al., 1999). Interestingly, it was also recently reported that increasing ANP levels with sacubitril, combined with RAAS blockage by valsartan has beneficial effects on kidney function and BP development in Dahl SS rats (Polina et al., 2020). The transcriptional regulation of ANP and BNP is believed to have two distinct arms; one which is turned on during development and another that is responsible for their upregulation in response to acute and chronic cardiac damage and stress (Man et al., 2018; Man et al., 2021; Sergeeva et al., 2016; Sergeeva & Christoffels, 2013). Thus, it is likely that this is a dynamic region of transcriptional regulation that adapts as needed during development or disease. Furthermore, this region contains a super enhancer, RE1, and a topologically associated domain (TAD) (Man et al., 2021). Genetic knockout of the RE1 region did not significantly affect *Clcn6* expression in ventricular or lung tissue; however, small changes in *Plod1* and *2510039O18Rik* were observed in ventricular tissue and *Mfn2* and *Agtrap* expression were decreased in the lungs, suggesting the potential for tissue specific alterations associated with mutations in this region (Man et al., 2021).

In this study, we examined whether loss of ClC‐6 function improves mortality rate and neuronal damage in Dahl SS rats on an 8% NaCl diet and if a mutation within this gene could influence *Mthfr*, *Nppa*, and *Nppb* expression. Although less commonly used than the stroke‐prone spontaneously hypertensive rats, Dahl SS rats placed on a high NaCl diet also experience hypertension‐induced blood–brain barrier disruption, stroke, and death, with cerebral hemorrhage and ischemic infarctions (Lin et al., 1999; Payne & Smeda, 2002; Werber et al., 1985). We found that loss of function mutation in *Clcn6* on the Dahl SS rat background does not convey protection against hypertension‐induced stroke mortality, potentially due to confounding altered expression of *Mthfr, Nppa*, and *Nppb*. This interesting result demonstrates the importance of coordinate genetic regulation, particularly in multifactorial diseases such as hypertension, and underscores the need to be mindful that not all genetic modifications represent a single altered variable in a disease model.

## 2 METHODS

### 2.1 Animals

The animal use and welfare procedures adhered to the National Institutes of Health (NIH) Guide for the Care and Use of Laboratory Animals following protocols reviewed and approved by the Medical College of Wisconsin (MCW) Institutional Animal Care and Use Committee. The strain of Dahl Salt‐Sensitive (SS) rats used in our studies (SS/JrHsdMcwi) has been inbred for more than 50 generations at MCW and is a well‐established model for salt‐sensitive hypertension. All rats were maintained in a standard 12/12 dark/light cycle and all tissue samples were collected between 11 am and 2 pm. All protocols used age‐matched (8–12‐week‐old male) SS‐WT and SS‐*Clcn6* (SS‐*Clcn6* ^*em2Mcwi*^ ) rats maintained on a custom AIN‐76 diet (Dyets, Inc.; 0.4% NaCl, #D113755). The SS‐*Clcn6* rats were generated previously via zinc finger nuclease (ZFN) mutagenesis, which causes a 15 base pair deletion in exon 13 and has previously been published (Flister et al., 2013; Klemens et al., 2021). To assess survival and stroke response in SS‐WT and SS‐*Clcn6* rats, animals were placed on an 8% NaCl diet (Dyets, Inc; #D100078) and followed until time of death or euthanasia was warranted as determined by paralysis, respiratory distress, seizures, and/or ataxia.

### 2.2 Plasma and urine measurements

Urine was collected for 24 h prior to initiating the HS diet and after 3 weeks on a HS diet in metabolic cages (40,615; Laboratory Products). When necessary, the rats were euthanized under isoflurane anesthesia, blood samples from the descending aorta were collected, and their kidneys were perfused with phosphate buffered saline (PBS). Tissues (kidneys, hearts, livers, and brains) were removed, placed in formalin or snap‐frozen in liquid nitrogen, and used in downstream procedures. Brains were isolated immediately after termination. Plasma and urine electrolyte concentrations were determined by the ABL800 FLEX blood gas analyzer (Radiometer America) as described previously (Palygin et al., 2019).

Urine microalbumin and creatinine levels were determined by an albumin blue fluorescent assay kit (Active Motif) and fluorescent plate reader (FL600, Bio‐Tek). Urinary creatinine levels were determined by an assay kit from Cayman Chemical. Albumin to Creatinine ratios (ACR) were determined by dividing microalbumin concentrations (mg/ml) by creatinine concentrations (mg/ml).

### 2.3 Histology

For histological and IHC analyses, tissues were submerged in formalin immediately following euthanasia and processed by the Children's Research Institute Histology Core as described previously (Palygin et al., 2019). Briefly, paraffin‐embedded tissues were cut into 4 μm (kidney) or 6 μm (brain) sections then dried, de‐paraffinized, and processed for hematoxylin and eosin staining, or immunohistochemical probing with an antibody directed against MTHFR (1:100; Invitrogen, Cat#: MA5‐15844, RRID:AB_11154428). Assessment of pyknotic nuclei was determined according to recommendations provided by Garman 2011, by a blinded experimenter (Garman, 2011).

### 2.4 TTC brain staining

2,3,5‐triphenyltetrazolium chloride (TTC) straining allows easy detection of injured brain tissue. After immediate removal, brains were placed in ice cold PBS, then transferred to a 1% TTC solution in PBS and incubated at 37°C for 2 h before washing with PBS and transferring to formalin for fixation and sectioning. In live, metabolically active tissue, TTC is oxidized by a mitochondrial dehydrogenase to a red pigment, formazan, whereas no reaction occurs in dead tissue, which remains white/pale.

### 2.5 Plasma homocysteine measurements

Total plasma homocysteine measurements were performed by a clinical laboratory using a stable isotope dilution microflow liquid chromatography tandem mass spectrometry approach.

### 2.6 RT‐qPCR

The qPCR analysis was done using previously described methods (Palygin et al., 2018, 2021). Briefly, according to the manufacturer's protocol, total RNA from flash‐frozen cortical kidney sections and heart apexes were isolated using Trizol Reagent (ThermoFisher). Total RNA quantity was determined by Nanodrop 2000 (ThermoFisher). The cDNA was generated using the RevertAid First Strand cDNA Synthesis Kit (ThermoFisher) with the random hexamer primer. Real‐time PCR reactions were carried out on an ABI Prism 7900HT (ABI, Applied Biosystems) using Bullseye EvaGreen qPCR Master Mix (MedSci). Per gene, multiple pairs of exon spanning primer sets were designed from the rat sequences of *Nppa*, *Nppb*, and *Mthfr* (Table 1). Best primer pairs were chosen by testing with different concentrations of the same cDNA sample before running the experimental samples. Quantification of *Nppa*, *Nppb*, and *Mthfr* mRNA was determined by normalizing to actin (*Actb*). Expression of *Nppa* and *Nppb* was quantified in cardiac tissue and *Mthfr* in kidney tissue.

**TABLE 1:** qPCR primers

Gene	Forward	Reverse
*Actb*	ctctgtgtggattggtggct	cgcagctcagtaacagtccg
*Nppa*	tctgatggatttcaagaacctgct	ttcatcggtctgctcgctca
*Nppb*	agttcaagctgctttgggca	taaaacaacctcagcccgtc
*Mthfr (all)*	agggcatcctcaccatcaac	gaactcgaggtaggccttctg
*Mthfr_*x1	aaccatcaaaaagccggggt	ggctggattcctgctgataga
*Mthfr_*x3	gatctgacgcatgagcaggaat	aactgtccttggaactctcgc

### 2.7 Statistical analysis

Results are presented as mean ± *SEM*, where N are independent animals and n are the number of replicates. Data were analyzed using GraphPad Prism 7 (GraphPad Software) and SigmaPlot 12.5 (SYSTAT Software). Data were tested for normality using Shapiro–Wilk test, then compared using one‐way ANOVA followed by Holm‐Sidak post‐hoc analysis, Kruskal–Wallis with Dunn's post‐hoc test, two‐way ANOVA with repeated measures, or unpaired two‐tailed student *t*‐test. Differences were considered statistically significant at *p* < 0.05.

## 3 RESULTS

### 3.1 Mortality is not improved in SS‐*Clcn6* rats

**FIGURE 1:**
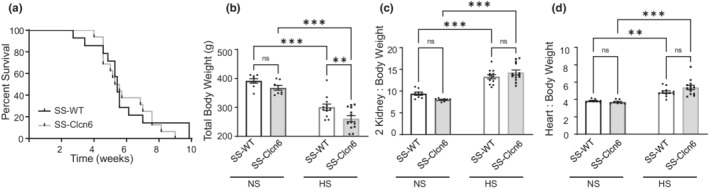
Survival and body measurements. (a) Kaplan Meier survival curve. The median survival for both SS‐WT (*N* = 14) and SS‐Clcn6 (*N* = 16) rats was 5.4 weeks. (b) Total body weights for age‐matched male SS‐WT and SS‐*Clcn6* rats on a normal salt (0.4% NaCl, NS) or high salt (8% NaCl, HS) diet, *N* ≥ 8 rats. (c) Kidney to body weight ratios, *N* ≥ 8 rats. (d) Heart to body weight ratios, *N* ≥ 8 rats. Data are presented as Mean ± *SEM*, One‐way ANOVA with Holm‐Sidak's multiple comparisons test determined significance (b–d), **p < 0.01, ***p < 0.001.

We tested whether SS‐*Clcn6* rats would have improved survival under chronic salt exposure compared to SS‐WT control rats. Rats were placed on an 8% NaCl (HS) diet and monitored over time. In many cases, due to the ethical concerns, euthanasia was warranted prior to endpoint, when apparent stroke symptoms were observed (limb paralysis, seizures, etc.). We found that there was no significant difference in survival between SS‐WT and SS‐*Clcn6* rats, and the median survival time for both groups was 5.4 weeks on a HS diet (Figure 1a). Physiological parameters of rats on the HS diet were also compared to age‐matched SS‐WT and SS‐*Clcn6* rats on a normal salt (NS) diet. There were no significant differences in total body weight or 2 kidneys to body weight and heart to body weight ratios in WT and SS‐*Clcn6* rats on an NS diet. As shown in Figure 1b, both SS‐WT and SS‐*Clcn6* rats had significantly decreased total body wieghts. (SS‐WT 391.5 ± 8.0 vs. 300.6 ± 10.8 g, N ≥ 8 rats, *p* < 0.0001; SS‐*Clcn6* 367.5 ± 8.2 vs. 238.3 ± 10.3 g, N ≥ 8, *p* < 0.0001) on a HS diet compared to their genetic group weights on a NS diet, and SS‐*Clcn6* rats had significantly decreased total body weights compared to SS‐WT rats on a HS diet (276.8 ± 10.8 vs. 238.3 ± 10.3 g, *N* ≥ 12 rats, *p* < 0.01). While both groups displayed significant increases in two kidneys to body weight and heart to body weight when comparing NS to HS diets, there were no significant differences between genotypes (Figure 1c,d).

We also measured plasma electrolytes of age‐matched rats on NS and HS diets, and urinary excretion values of SS‐WT and SS‐*Clcn6* at baseline on a NS diet, and after 3 weeks of HS diet. While the HS diets resulted in significant changes in Na^+^ and K^+^ plasma electrolytes, there were no significant differences between genotypes on either diet (Table 2). Similarly, while altered compared to the NS diet values, changes in 24 h urine volume, Na^+^, Cl^−^, and creatinine excretion were not significant between SS‐WT and SS‐*Clcn6* rats after 3 weeks of HS diet (Table 3).

**TABLE 2:** Plasma Electrolytes. Mean electrolyte concentrations ± *SEM* from SS‐WT or SS‐Clcn6 rats on normal salt (0.4% NaCl, NS) or high salt (8.0% NaCl, HS) diets. Plasma K^+^ was significantly reduced and Na^+^ significantly increased in both SS‐WT and SS‐Clcn6 rats on a HS diet; however, there were no significant differences between genotypes. *p < 0.05 between diets as determined by Kruskal–Wallis one‐way ANOVA corrected for multiple comparisons with Dunn's post‐hoc analysis

Electrolyte (mM)	NS		HS	
	SS‐WT (*N* = 8)	SS‐Clcn6 (*N* = 8)	SS‐WT (*N* = 11)	SS‐Clcn6 (*N* = 10)
K^+^	3.9 ± 0.1	3.8 ± 0.1	3.1 ± 0.2*	3.1 ± 0.1*
Na^+^	140.6 ± 0.9	139.0 ± 0.4	145.1 ± 0.9*	145.9 ± 1.5*
Ca^2+^	1.32 ± 0.02	1.34 ± 0.01	1.30 ± 0.04	1.29 ± 0.06
Cl^−^	108.4 ± 1.0	105.3 ± 1.5	103.7 ± 1.4	107.8 ± 2.2

**TABLE 3:** Urinary Values. 24 h urine volumes, creatine excretion, electrolyte, and albumin concentrations normalized to creatinine. Two‐way repeated measures ANOVA was used to analyze data between groups. While there were significant increases in urine volume, Na^+^, Cl^−^, Ca^2+^, and albumin excretion as well as significantly decreased creatinine on day 21 of 8% NaCl diet, there were no significant differences between SS‐WT and SS‐Clcn6 on Day 0 or Day 21. Normal salt (0.4% NaCl, NS) or high salt (8.0% NaCl, HS). Data shown is the mean ± *SEM*. *p < 0.05 between diets

24 h urinary values	Day 0		Day 21	
	SS‐WT (*N* ≥ 5)	SS‐Clcn6 (*N* ≥ 6)	SS‐WT (*N* ≥ 5)	SS‐Clcn6 (*N* ≥ 6)
Volume (ml/24 hrs)	10.6 ± 3.5	8.3 ± 1.0	51.8 ± 8.5*	41.6 ± 7.0*
Creatinine (mg/dl)	80.0 ± 22.6	84.6 ± 24.6	17.5 ± 3.8*	16.1 ± 2.6*
K^+^/Cre	16.3 ± 1.3	15.2 ± 0.7	14.3 ± 1.1	14.3 ± 0.6
Na^+^/Cre	9.4 ± 1.2	14.1 ± 0.6	188.8 ± 34.3*	186.8 ± 25.7*
Ca^2+^/Cre	0.09 ± 0.02	0.11 ± 0.01	1.22 ± 0.22*	1.25 ± 0.25*
Cl^−^/Cre	23.8 ± 2.8	23.3 ± 1.6	174.0 ± 30.1*	168.2 ± 20.8*
Alb/Cre	1.27 ± 0.20	0.78 ± 0.20	19.9 ± 1.7*	20.2 ± 3.4*

### 3.2 Neuronal cell death is comparable between WT and SS‐*Clcn6* rats

**FIGURE 2:**
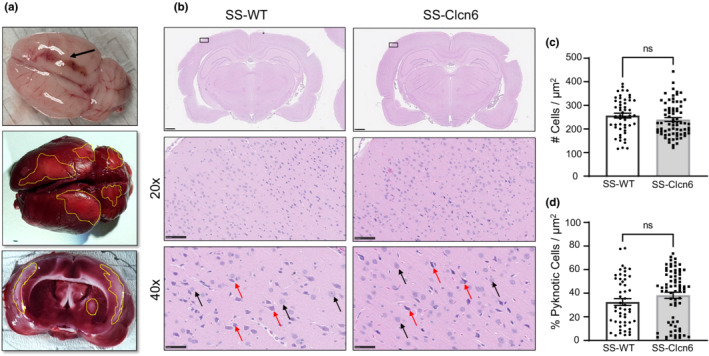
Neuronal Cell Death. (a) Brains from rats on high salt diets were assessed for gross structural damage following immediate removal after euthanasia (top, see necrotic area indicated by arrow). Verification and localization of neurological damage was assessed with 2,3,5‐triphenyltetrazolium chloride (TTC) staining. Active metabolic areas (live tissue) metabolize TTC to a red color, whereas dead tissue remains white‐pink (yellow outlines). (b) H&E‐stained coronal brain sections of SS‐WT and SS‐Clcn6 rats at 1x, 20x, and 40x magnification (scale bars are 1, 100, and 50 μm, respectively). Arrows indicate representative healthy neurons (black) and pyknotic neurons (red). (c) Graph summarizing the total number (healthy and pyknotic) of neurons per area. (d) Summarized data of the percentage of dying (pyknotic) neurons per area. *N* = 5 rats, *n* = 6 fields of view at 40× from cortex. Data are presented as Mean ± *SEM*, unpaired student's two‐tailed *t*‐test was used to determine significance.

Previous work found severe pathological changes in the brains of Dahl SS rats on very high salt diets, which included hemorrhage, edema, and infarction (Dene & Rapp, 1985; Werber et al., 1985). We utilized 2,3,5‐triphenyltetrazolium chloride (TTC) staining in several SS‐WT and SS‐Clcn6 rats in addition to gross assessment to determine common areas of brain damage (Figure 2a), which was consistently observed in the motor and somatosensory cortex as well as occasionally in the thalamus in response to prolonged HS exposure. This was consistent with frequent forelimb paralysis that was observed in many animals. To determine whether neurological damage might be different between SS‐WT and SS‐*Clcn6* rats, we used coronal sections stained with hematoxylin and eosin and counted the total number of neuronal cell bodies and neurons undergoing cell death (pyknotic) in the motor cortex. Pyknotic neurons were identified by changes in shape and color by blinded image analysis (Figure 2b). There were no significant differences in the total number of neurons per area or the % of pyknotic nuclei per area (Figure 2c,d, *N* = 5–6 rats, *n* ≥ 6 fields of view per rat).

### 3.3 Loss of ClC‐6 results in altered mRNA and protein expression of methylenetetrahydrofolate reductase (MTHFR)

**FIGURE 3:**
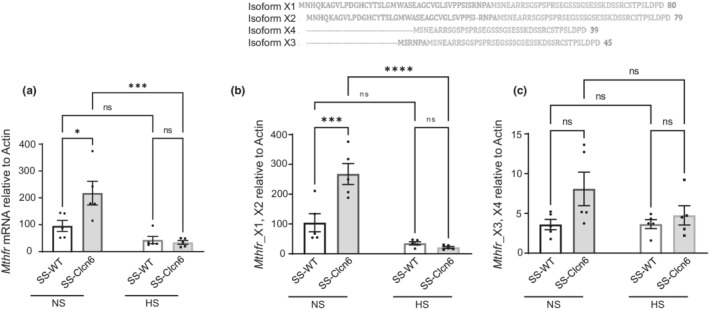
Methylenetetrahydrofolate reductase (*Mthfr*) mRNA expression. (a) Quantitative PCR analysis of *Mthfr* mRNA expression normalized to actin in SS‐WT or SS‐*Clcn6* rats on a normal salt (NS) or high salt (8% NaCl, HS) diet, *N* = 5 rats. The gene encoding *Mthfr* has 2 start codons in close proximity to *Clcn6*, so primers were designed to identify the (b) longer (X1, X2) and (c) shorter (X3, X4) isoforms of *Mthfr* and quantify their expression. Data are presented as Mean ± *SEM*, significance determined by one‐way ANOVA with Holm‐Sidak's multiple comparisons test, *p < 0.05, **p < 0.01, ***p < 0.001.

The gene encoding *CLCN6* is located in an AGTRAP‐PLOD1 gene cluster conserved between humans and rats. This cluster contains several genes known to contribute to blood pressure regulation and cardiovascular health (Del Greco et al., 2011; Flister et al., 2013; Gaughan et al., 2000; Man et al., 2018). In particular, *CLCN6* is flanked by *NPPA*, *NPPB*, and *MTHFR*, and in proximity to CCCTC‐binding factor (CTCF) binding sites believed to influence both *NPPA* and *NPPB* expression (Del Greco et al., 2011; Sergeeva et al., 2016). First, we assessed whether loss of *Clcn6* in our knockout model could affect renal expression of *Mthfr* using RT‐qPCR. As shown in Figure 3a, we found that on a NS diet, SS‐*Clcn6* rats had significantly increased *Mthfr* expression compared to SS‐WT rats (95.6 ± 20.4 vs. 217.80 ± 44.0 a.u., *N* = 5, *p* < 0.05). HS diets promote a reduction in *Mthfr* expression in both strains with the significant difference in the SS‐*Clcn6* (Figure 3a, 217.8 ± 44.0 vs. 34.1 ± 7.0 a.u., NS vs. HS; *N* = 5, *p* < 0.001). Since there are different transcription start sites and alternative splicing that can occur in *Mthfr* (Tran et al., 2002), we also generated distinct primers to pick up the isoforms with different start codons, and found that the longer isoform more closely followed the pattern seen in the primer set which identified all isoforms (Figure 3b,c).

**FIGURE 4:**
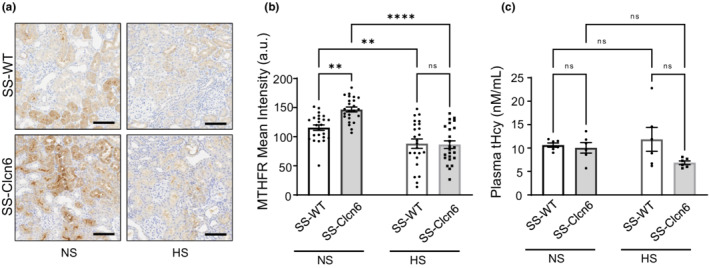
MTHFR renal protein expression and circulating homocysteine levels. (a) Representative images of MTHFR IHC in the kidney in WT and knockout rats on both diets. Scale bars are 100 μm. (b) Summarized data of MTHFR protein quantification in kidneys *N* = 6 rats, *n* = 8 fields of view at 20× per rat, cortex region. (c) Total plasma homocysteine (tHcy) levels. Data are presented as Mean ± *SEM*, significance determined by one‐way ANOVA with Holm‐Sidak's multiple comparisons test, *p < 0.05, **p < 0.01, ***p < 0.001.

MTHFR protein expression was also assessed using immunohistochemistry in paraffin‐embedded kidney sections and quantified by color thresholding (Figure 4a,b). Similar to the RT‐qPCR results, SS‐*Clcn6* rats had significantly increased MTHFR expression on a NS diet, and the HS diet resulted in a reduction in protein expression in both strains. To further interrogate the observed differences in the *Mthfr* gene, we measured circulating plasma homocysteine (tHcy) levels as an indicator of systemic MTHFR activity. MTHFR is a key enzyme responsible for breaking down homocysteine. There were no significant differences between genotypes or diets (Figure 4c).

### 3.4 SS‐*Clcn6* rats display distinct differences in *Nppa* and *Nppb* mRNA expression

**FIGURE 5:**
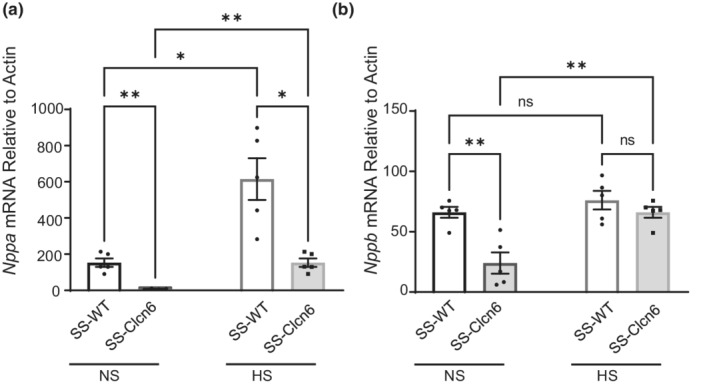
Cardiac expression of *Nppa* and *Nppb*. (a) Quantitative *Nppa* mRNA expression normalized to actin from age‐matched SS‐WT and SS‐*Clcn6* rats on a normal salt (NS, 0.4% NaCl) or high salt (8% NaCl, HS) diet. (b) Quantitative *Nppb* mRNA expression normalized to actin from age‐matched SS‐WT and SS‐*Clcn6* rats on a NS or HS diet. *N* = 6 rats, data are presented as Mean ± SEM, significance determined by one‐way ANOVA with Holm‐Sidak's multiple comparisons test, **p < 0.01, ***p < 0.001.

To look more closely at discordant gene regulation resulting from a mutation in *Clcn6*, we also assessed *Nppa* and *Nppb* expression in cardiac tissue. In contrast to the *Mthfr* gene, mRNA expression of both *Nppa* (153.0 ± 23.3 vs. 17.61 ± 10.7 a.u., *N* = 5, *p* < 0.001) and *Nppb* (66.0 ± 4.5 vs. 24.0 ± 8.8 a.u., *N* = 5, *p* < 0.01) was significantly lower in SS‐*Clcn6* rats on the NS diet (Figure 5a). Moreover, *Nppa* expression in both SS‐WT and SS‐*Clcn6* was significantly augmented on the HS diet, but still was significantly lower in SS‐*Clcn6* group (614.9 ± 115.2 vs. 153.0 ± 23.3, *N* = 5, *p* < 0.001) (Figure 5a). Cardiac expression of *Nppb* in knockout rats significantly increased on the HS diet. In contrast, WT expression did not change (Figure 5b).

## 4 DISCUSSION

Garnering new information about novel genes involved in BP regulation is essential to further our understanding of stroke and hypertension. In the present study, we postulated that genetic ablation of *Clcn6* on the Dahl SS rat background might improve mortality and pathophysiological damage induced by an 8% NaCl diet. However, the median survival rate and neurological damage did not differ between SS‐WT and SS‐*Clcn6* rats. Further interrogation of our model revealed that a 15 base pair deletion in *Clcn6* was associated with expression changes in nearby genes, *Mthfr*, *Nppa*, and *Nppb*. This altered regulation is important for understanding the complexity of pathophysiological processes since all 3 of these genes are involved in BP regulation.

Given the similarity in survival, it is not surprising that we did not observe significant differences in neuronal cell death or number. Identification of pyknotic nuclei from an H&E stain is less accurate than Fluro‐Jade and caspase cleavage, and it is possible more subtle differences would have been detected using these methods. It may have been interesting to examine differences in microglia, astrocytes, and inflammatory cells; however, given the lack of difference in mortality, we did not anticipate observing major changes. Worth noting is that while SNPs in *CLCN6* are associated with lower DP, they have also been found to be associated with seizure and epilepsy in some populations (Wang et al., 2017; Yamamoto et al., 2015), and a *Clcn6* deficient mouse had characteristics of mild neuronal ceroid lipofuscinosis, a lysosomal storage disease (Poet et al., 2006). Furthermore, a recent study from Polovitsksaya et al, identified a de novo missense gain‐of‐function mutation in *CLCN6* that is associated with an early‐onset neurodegenerative disorder presenting with developmental delay, severe generalized hypotonia, respiratory insufficiency, neurogenic bladder, and cerebral atrophy (Polovitskaya et al., 2020). In neurons, ClC‐6 protein functions as an endolysosomal Cl^−^/H^+^ exchanger (Neagoe et al., 2010), and additional differences in lysosomal clearance during the development of hypertension could potentially affect cerebral damage in ways distinct from SS‐WT rats.

*MTHFR* encodes methylenetetrahydrofolate reductase, an important enzyme in the folate‐methionine metabolic cycle that breaks down homocysteine. Numerous studies have found that polymorphisms in *MTHFR* are associated with hyperhomocysteinemia, essential hypertension, and stroke (Meng et al., 2021; Wei et al., 2017). The kidneys are a critical player in the regulation of plasma Hcy levels, and remove ~70% of the daily Hcy load (Refsum et al., 1998). In the knockout rats, we observed significantly increased *Mthfr* expression at both the RNA and protein levels in the kidney, which would be predicted to be protective against stroke. Additionally, if this crosstalk between the genes is similar in humans, it may in part explain how the loss of function mutations in *CLCN6* are associated with the “long survivor” sickle cell anemia patient cohort. In this case, patients with loss of *CLCN6* function would have significantly increased MTHFR and will be more able to efficiently break down pathologically elevated homocysteine (Wonkam et al., 2020). When we tested the tHcy in all four conditions, we did not observe any significant differences; however, this may be due to an underpowered sample size and the large variability of time from which the end‐stage plasma samples were collected from the rats on the 8% NaCl diet. For the HS samples that were tested, the available plasma samples varied from 5 to 8 weeks of 8% diet. The kidney is responsible for Hcy extraction and metabolism, and circulating Hcy levels can be impacted by glomerular filtration rate (GFR) and renal damage (Friedman et al., 2001). Hyperhomocysteinemia in kidney disease is partially attributable to defective Hcy clearance, and hemodialysis can acutely reduce Hcy levels (House AA et al., 2000). Hyperhomocysteinemia is believed to be a contributor to glomerular damage and fibrosis deposition in salt‐sensitive hypertension (Li et al., 2002). The normotensive tHcy values (10.6 ± 1.1 and 10.0 ± 1.1 μM, SS‐WT and SS‐*Clcn6* respectively) were consistent with previously reported values (Li et al., 2006). Lastly, Hcy levels are influenced by GFR, and ANP can also regulate GFR, so the downregulation in *Nppa* may also be influencing the tHcy measurements.

While the increased MTHFR expression may play a protective role in the SS‐*Clcn6* rats, the reduction in ANP and BNP would have the opposite effect; namely, the SS‐*Clcn6* rats would have a diminished ability to vasodilate and reduce Na^+^ and water excretion, thereby impairing regulation of intravascular volume and pressure. Renal ANP signaling has important ramifications for Na^+^ and water homeostasis (Theilig & Wu, 2015), and while we did not observe a significant difference in Na^+^ excretion between WT and knockout rats on an 8% NaCl diet, this is potentially due to the excessive sodium load. A ~50% reduction in Na^+^ excretion was previously reported for the SS‐*Clcn6* rats after 10 days on the lower 4% NaCl diet (Flister et al., 2013). Since ClC‐6 is also found in the proximal tubule, thick ascending limb, distal convoluted tubule, and collecting duct according to a nephron segment‐specific deep sequencing database (Lee et al., 2015; Zhao et al., 2016), it would be of interest to more closely examine the expression of Na^+^ transporters and channels in these regions. ANP inhibits several Na^+^‐dependent transport systems, including the Na^+^‐H^+^ exchanger (Winaver et al., 1990), type IIa Na‐Pi cotransporter (Bacic et al., 2001), Na^+^‐K^+^‐ATPase (Aperia et al., 1994), Na^+^‐K^+^‐2Cl^−^ cotransporter 2 (Ares et al., 2011), and the epithelial sodium channel (ENaC) (Guo et al., 2013; Wang et al., 2006). In the kidney, the collecting duct is a main target of ANP (Inoue et al., 2001), which acutely reduces ENaC activity through a reduction in open probability through a cGMP specific signaling pathway and increases surface expression of the α‐ and γ‐ENaC subunits within 90 minutes of ANP stimulation (Guo et al., 2013; Wang et al., 2006). Additionally, ANP acts as a counterbalance to the RAAS system, acting as an antagonist of Angiotensin II signaling and exerting oppositional effects systemically. With reduced ANP expression in both normotensive and hypertensive conditions, the SS‐*Clcn6* rats may exhibit a distinct RAAS metabolite profile, which could further alter their distal nephron Na^+^ and water transport capacities.

Future studies should verify the circulating ANP and BNP levels in normotensive and hypertensive rats in the WT and knockout animals and perform more sophisticated omics analysis and chromatin remodeling assays to validate these observations. In our previous study, we did observe a lower diastolic (but not systolic) BP at baseline, and this could be contributing to the differences in *Nppa* and *Nppb* expression, despite no differences in the assessed cardiac functions at baseline.

This study provides evidence of how a complex genetic regulatory landscape can influence research outcomes. In attempting to understand the role of *CLCN6* in stroke, we have additionally discovered that a mutation in this conserved region is associated with altered expression of nearby blood pressure homeostasis genes. Specifically, in our case, a mutation causing a dysfunctional ClC‐6 protein, results in increased *Mthfr*, which should potentially be protective; however, it also reduces *Nppa* and *Nppb* expression, which would be predicted to be deleterious. These changes could have widespread effects that are interrelated to one‐another. Furthermore, this model serves as an important example of how genes do not exist in isolation and conserved regulatory networks may need more than simple knockout models to fully understand their physiological impact.

## AUTHOR CONTRIBUTIONS

CAK, OP, and AS designed the study; CAK, LD, AZ, and VL performed the investigations; CAK, OP, LD, and AZ analyzed the data; CAK made the figures; CAK, OP, and AS drafted and revised the paper. All authors approved the final version of the manuscript.

## FUNDING INFORMATION

This work was supported by Department of Veteran Affairs Grant I01 BX004024 (to AS), National Institutes of Health Grants K99 HL153686 (to CAK), R35 HL135749 (to AS), R01 DK126720 (to OP), T32 HL134643 (CVC A.O. Smith Fellowship to CAK), and UL1 TR001450/SCTR 2214 (to OP).

## CONFLICT OF INTEREST

None.
